# Whole transcriptome analysis reveals differential gene expression associated with *Anaplasma phagocytophilum* invading HL-60 cells

**DOI:** 10.1186/s13071-026-07381-6

**Published:** 2026-05-08

**Authors:** Ke Shi, Lulu Sun, Shuxing Qiu, Yankai Chang, Zi Yan, Yaqun Yan, Fuchun Jian, Junqiang Li, Rongjun Wang, Longxian Zhang, Changshen Ning

**Affiliations:** 1 School of Medicine, Xinxiang University, Jinsui Road 191, Xinxiang, 453003 China; 2 College of Veterinary Medicine, Henan Agricultural University, Zhengzhou, 450046 China; 3 International Joint Research Laboratory for Zoonotic Diseases of Henan, Zhengzhou, 450046 China

**Keywords:** A. hagocytophilum, HL-60 cells, RNA-seq, NcRNAs, Competing endogenous RNA

## Abstract

**Background:**

*Anaplasma phagocytophilum* is an obligate intracellular, tick-borne bacterial pathogen capable of causing disease and even mortality in various mammals, including humans. Non-coding RNAs play important regulatory roles in multicellular organisms, including innate and adaptive immune pathways, which control bacterial, parasitic, and viral infections. However, the global transcriptomic landscape encompassing both ncRNAs and mRNAs in HL-60 cells invaded by *A. phagocytophilum* remains unexplored.

**Methods:**

Cell apoptosis was evaluated by flow cytometry at multiple time points after HL-60 cell infection with *A. phagocytophilum*. Total RNA was extracted and analyzed by RNA sequencing (RNA-seq) to delineate expression alterations of long non-coding RNAs (lncRNAs), microRNAs (miRNAs), and messenger RNAs (mRNAs) at 24 h post-infection (hpi). Bioinformatics methods were employed for gene ontology (GO) and Kyoto Encyclopedia of Genes and Genomes (KEGG) pathway enrichment analyses to elucidate the potential functions of these differentially expressed genes. Furthermore, an integrated bioinformatics approach was applied to systematically construct a competing endogenous RNA (ceRNA) network involving lncRNAs, miRNAs, and mRNAs.

**Results:**

*A. phagocytophilum* infection accelerated HL-60 cell apoptosis at multiple time points, with the most significant effect observed at 24 hpi. Transcriptome profiling at 24 hpi identified substantial differential expression, including 487 lncRNAs, 550 mRNAs, and 22 miRNAs with statistically significant changes in expression. Then, expression patterns of eight lncRNAs, eight mRNAs, and seven miRNAs were experimentally validated through reverse transcription quantitative polymerase chain reaction (RT-qPCR), demonstrating strong correlation with RNA-seq results. Bioinformatics analyses revealed significant enrichment of differentially expressed mRNAs in three key pathways: the PI3K/Akt signaling pathway, the actin cytoskeleton regulation pathway and the p53 signaling pathway. Differentially expressed lncRNAs were largely related to the phospholipase D signaling pathway and pathways related to cortisol and aldosterone synthesis/secretion. The altered miRNAs showed predominant enrichment in Rap1 and NF-κB signaling pathways. Notably, computational reconstruction of the lncRNA–miRNA–mRNA ceRNA network identified hsa–miR–4518 and hsa–miR–3609 as central regulatory nodes.

**Conclusions:**

This comprehensive transcriptome study elucidates complex gene regulatory networks activated in HL-60 cells after *A. phagocytophilum* invasion, with particular emphasis on pathogen-modulated miRNA signatures that coordinate critical pathways governing host immune responses and microbial survival strategies. These findings elucidate previously uncharacterized molecular mechanisms underlying *A. phagocytophilum* pathogenesis and may provide actionable targets for novel therapeutics.

**Graphical Abstract:**

**Figure:**
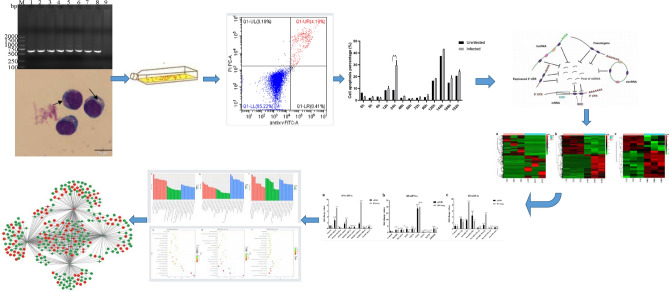
Whole Transcriptome Analysis RevealsDifferential Gene Expression in HL-60 Cells Invaded by *Anaplasma phagocytophilum*

**Supplementary Information:**

The online version contains supplementary material available at 10.1186/s13071-026-07381-6.

## Background

*Anaplasma phagocytophilum* is a tick-borne pathogen of substantial medical and veterinary importance. This bacterium replicates within membrane-bound cytoplasmic vacuoles in both vertebrate host neutrophils and tick vector cells, causing granulocytic anaplasmosis with varying clinical manifestations across multiple host species, ranging from human granulocytic anaplasmosis (HGA) in humans to tick-borne fever in domestic animals [1–5]. HGA typically presents with nonspecific systemic symptoms such as fever, headache, and myalgia [4]. However, treatment delays or underlying medical conditions may lead to life-threatening complications, including respiratory failure, gastrointestinal hemorrhage, acute kidney injury, hepatic dysfunction, and fatal outcomes [4, 6]. From 2001 to 2015, more than 15,000 HGA cases were reported in the USA [7]. However, the reported incidence of HGA in Europe is relatively low, with case numbers not surpassing 300 [7]. In Asia, HGA has been reported in China, Japan, and South Korea [8]. Notably, a Chinese surveillance study (2009–2010) identified 46 confirmed and 16 suspected human cases with an 8.1% fatality rate [9]. Concurrently, high *A. phagocytophilum* infection rates were detected in local ruminants, including yaks (32.3%), sheep (42.1%), and cattle (41.08%) [9–11], highlighting the zoonotic reservoir potential. To establish infection, *A. phagocytophilum* employs sophisticated strategies such as cytoskeleton remodeling, apoptosis inhibition, immune modulation, and antigenic variation [12–15]. Despite these advances, major challenges remain in developing clinically effective vaccines and rapid diagnostic tests with practical utility.

Non-coding RNAs (ncRNAs) serve as key regulatory factors of transcriptional and post-transcriptional processes, participating in diverse biological functions [16]. Long non-coding RNAs (lncRNAs), defined as ncRNAs exceeding 200 nucleotides, play essential roles in chromatin modification, epigenetic regulation, cell cycle control, apoptosis, and differentiation [17, 18]. Additionally, microRNAs (miRNAs) are a highly conserved class of small non-coding RNAs (~18–22 nucleotides) that regulate gene expression post-transcriptionally through sequence-specific binding to the 3′ untranslated region (3′ UTR) of target mRNAs [19]. Recent studies have revealed that *A. phagocytophilum* alters the miRNA expression profile in *Ixodes scapularis* tick cells, notably upregulating isc-miR-79, which facilitates bacterial infection through the Robo2 signaling pathway [5]. Repression of tick microRNA-133 induces organic anion transporting polypeptide expression critical for *A. phagocytophilum* survival in the vector and transmission to the vertebrate hosts [20]. Understanding the regulatory roles of the host non-coding RNAs in pathogen infection is therefore crucial for elucidating host–pathogen molecular interactions and developing novel therapeutic strategies.

Furthermore, a comprehensive analysis of differential non-coding RNA expression in HL-60 cells following *A. phagocytophilum* invasion remains unexplored. This study systematically characterized the differential expression profiles of lncRNAs, miRNAs, and mRNAs in *A. phagocytophilum*-infected HL-60 cells and analyzed their associated functional pathways. By establishing a competing endogenous RNA (ceRNA) network, we further evaluated their biological roles in the infection process. These findings provide a foundation for elucidating the control mechanisms of host non-coding RNAs (ncRNAs) during *A. phagocytophilum* infection.

## Methods

### HL-60 cell and blood sample preparation

HL-60 cells (human acute promyelocytic leukemia cell line; ATCC CCL-240) were obtained from the Cell Bank of the Chinese Academy of Sciences (Beijing, China). The cells were cultured in Roswell Park Memorial Institute (RPMI) 1640 medium (the Cell Bank of the Chinese Academy of Sciences, Beijing, China) supplemented with 10% fetal bovine serum (FBS; Gibco) and 2 mM L-glutamine, and maintained at 37 °C in a humidified atmosphere containing 5% CO_2_. The *A. phagocytophilum* isolate was initially obtained from fresh blood collected from a naturally infected goat (accession number OL678411) exhibiting characteristic clinical signs, including pyrexia and mucosal pallor, at a farm in Luoyang, Henan Province, China [21]. Both 2 mL of *A. phagocytophilum*-positive fresh blood and 6 mL of red blood cell (RBC) lysis solution (Solarbio, China) were mixed in a 10-mL sterile centrifuge tube. The tube was then incubated in an ice bath for 15 min and gently inverted three times during incubation until the solution became clear. The mixture was centrifuged at 450 rpm at 4 °C for 10 min, the supernatant was removed, and two washes were performed on the pellet with 5 mL RPMI 1640 medium. Finally, the pellet was resuspended in 1 mL of RPMI 1640 medium.

A 200μL of granulocytes suspended in RPMI 1640 medium was added to a 5 mL culture of 2 × 10^5^–6 × 10^5^ HL-60 cells/mL at 37 °C in a humidified 5% CO_2_ incubator. Infection progression was assessed daily through microscopic examination of Giemsa-stained preparations from 200 μL of HL-60 cells pellets infected by *A. phagocytophilum*. Microscopic examination (Leica DM5000) of 100 cells per slide, and cell cultures were harvested upon reaching an infection rate of > 90% [22].

### Cell-free bacteria preparation and sample collection

To selectively lyse HL-60 cells while preserving bacterial viability, the harvested cell suspension was mechanically lysed by six passes through a 27-gauge needle-equipped syringe. Then, the cellular debris was removed by centrifugation at 700 × g for 8 min, followed by high-speed centrifugation of the supernatant at 5000 × g for 15 min to sediment cell-free bacteria. The resulting pellet was resuspended in 300 μL of culture medium [22, 23]. Then, 100 μL of the cell-free bacterial suspension was added to 5 mL culture of 2 × 10^5^–6 × 10^5^ HL-60 cells/mL, designated as the treatment group, and uninfected HL-60 cells served as the control group. Each of the treatment and control groups included triplicate samples.

### Flow cytometry for HL-60 cell apoptosis

Apoptosis in HL-60 cells was evaluated using a fluorescein isothiocyanate (FITC)-conjugated Annexin V apoptosis detection kit (BD Biosciences Pharmingen, USA), strictly following the manufacturer’s instructions. For both the infected and uninfected control groups, approximately 5 × 10^5^ cells per sample were collected at 0, 3, 6, 12, 24, 48, 60, 72, 96, and 144 h post-infection. Cell counting was performed using a hemocytometer to ensure consistent cell numbers across samples. Harvested cells were washed twice with cold phosphate-buffered saline (PBS; pH 7.4) and resuspended in 200 μL of ice-cold 1× binding buffer. The cells were then stained with 10 μL of Annexin V-FITC, gently vortexed, and incubated on ice for 30 min in the dark. After incubation, 300 μL of ice-cold 1× binding buffer and 5 μL of propidium iodide (PI) were added to each sample. Apoptotic activity was immediately quantified using a flow cytometer (Beckman Coulter, Brea, CA, USA). Infected and control groups were processed in parallel at each time point to ensure consistent experimental conditions.

### Specimen acquisition, RNA isolation, and quality monitoring

Both *A. phagocytophilum*-infected (treatment) and uninfected (control) HL-60 cell samples (each sample contained cells within the target range of 5 × 10^5^–1 × 10⁶) were collected at 24 hpi. Total RNA was isolated in triplicate biological replicates with Trizol Reagent (TaKaRa, Dalian, China) as recommended by the manufacturer. RNA concentration and purity were assessed using the RNA 6000 nano kit (Agilent, Technologies, Santa Clara, CA, USA), while integrity was evaluated through both agarose gel electrophoresis and the Agilent 2100 Bioanalyzer (Agilent Technologies, Santa Clara, CA, USA).

### Library construction and whole transcriptome profiling

LncRNA and small RNA libraries were constructed separately. To construct the lncRNA library, ribosomal RNA (rRNA) was removed using the rRNA Depletion Module (H/M/R) Removal Kit (Abclonal, China). After purification, the ribosomal-RNA-depleted samples were hybridized with the probe at a high temperature of 95 ℃. First-strand complementary DNA (cDNA) was subsequently generated through reverse transcription with random primers (using the RNA Directional Library Preparation Kit, TaKaRa, Dalian, China). Subsequently, the second-strand cDNA synthesis was performed using DNA polymerase I and RNase H to generate double-stranded cDNA. The resulting cDNA was then subjected to A-tailing and adapter ligation. The ligated products were then amplified by polymerase chain reaction (PCR) using the following thermal cycling conditions: 12 cycles of 98 °C for 15 s, 60 °C for 30 s, and 72 °C for 30 s. Then the amplicons were thermally denatured to yield single-stranded DNA, and the single-stranded DNA was circularized using a bridge primer to form a single-stranded circular DNA library. Finally, each sample was diluted to 2 nM for paired-end sequencing on an Illumina HiSeq platform.

Small RNA libraries were prepared through the VAHTS Small RNA Library Prep Kit (Personalbio Technology Co., Ltd., China) and subsequently sequenced in single-end mode on an Illumina HiSeq platform. The construction included the following two main steps: initially, 3′ and 5′ adapters were ligated to the small RNAs using T4 RNA ligase and subsequently, double-stranded cDNA was synthesized using Superscript II reverse transcriptase (TaKara, Kyoto, Japan). Then, the enrichment of DNA fragments was accomplished by PCR with the addition of index parts and sequencing connectors. The target fragment product was isolated by 15% polyacrylamide gel electrophoresis (PAGE), and the library was purified according to the fragment size. Subsequently, the PCR-enriched fragments were analyzed using the Agilent 2100 Bioanalyzer (Agilent Technologies, Santa Clara, CA, USA) to evaluate the fragment size distribution and verify the library integrity for quality control purposes. The total and effective concentrations of the library were quantified using PicoGreen fluorescence assay and quantitative PCR (qPCR) (Takara Bio, Dalian), respectively. Finally, each sample was diluted to 10 nM and adjusted to a final concentration of 4–5 pM for sequencing on the Illumina HiSeq platform.

### Data quality assessment

The lncRNA library was sequenced on the Illumina HiSeq platform, yielding a high-throughput dataset referred to as “raw data.” To ensure the precision of downstream analyses, the initial sequencing outputs underwent stringent quality filtering to generate high-quality clean data. The FASTX-toolkit (http://hannonlab.cshl.edu/fastx_toolkit/) was used to eliminate the 3′ end joint sequence and the average mass fraction below a quality score (Q) 20 reads, filtering the raw reads to obtain the clean reads. The high-quality filtered reads, known as “clean reads,” were stored in FASTQ format [24]. The clean reads were subsequently aligned to the reference genome (Homo_sapiens.GRCh38.dna.primary_assembly.fa) using the HISAT2 software [25].

Similarly, clean tags of the small RNA library sequence were obtained by filtering out sequences that met any of the following criteria: (1) containing two or more low-quality bases (*Q*-value ≤ 20), (2) including ambiguous nucleotides (N), and (3) lacking a small RNA insert between the 3′ and 5′ adapters, or having a length of fewer than 18 nucleotides. These high-quality reads, termed “clean reads,” were archived in FASTQ format for downstream analysis [24]. Subsequently, the filtered reads were aligned to the reference genome using HISAT2 for transcript mapping and Bowtie (v.1.2.2) for miRNA mapping, as previously reported [25, 26].

To evaluate monotonic associations among samples, Spearman’s rank correlation analysis was conducted using R software (version 3.5.1) (Additional file 12: Fig. 8).

### Identification and screening of dif-lncRNAs, dif-mRNAs, and dif-miRNAs

Whole transcriptome profiling was conducted using the Illumina HiSeq platform, generating comprehensive data encompassing lncRNA, miRNA, and mRNA. Transcript abundance of both lncRNAs and mRNAs was measured in fragments per kb per million reads (FPKM) [27], while miRNA expression levels were assessed using transcripts per million (TPM) [28]. Differential expression analysis of mRNAs, lncRNAs, and miRNAs was performed using the DESeq software package (version 1.18.0), which models count data based on a negative binomial distribution [29]. Statistical significance was evaluated using the Wald test. To account for multiple hypothesis testing, *P*-values were adjusted using the Benjamini–Hochberg false discovery rate (FDR) method. Transcripts with an adjusted *P*-value (FDR) < 0.05 and |log2 fold change|≥ 1 were defined as differentially expressed. The expression profiles of differentially expressed lncRNAs (dif-lncRNAs), differentially expressed mRNAs (dif-mRNAs), and differentially expressed miRNAs (dif-miRNAs) were visualized using hierarchical clustering and volcano plots generated with the ggplot2 (https://github.com/tidyverse/ggplot2) in R package.

### Reverse transcription quantitative polymerase chain reaction

The reverse transcription qPCR (RT-qPCR) assays were implemented following the manufacturer’s protocol using SYBR Premix Ex Taq I (Takara Bio, Dalian). *GAPDH*, *β-actin*, and *U6* acted as internal reference genes for lncRNA, mRNA, and miRNA quantification, respectively. The reaction procedures described in the manual included an initial denaturation at 95 °C for 5 min followed by 40 amplification cycles of 95 °C for 20 s and 60 °C for 30 s, and a last extension at 72 °C for 20 s. The experiment was repeated three times. Sequences of primers are provided in Additional file 1: Table S1.

### Target gene identification for dif-lncRNAs and dif-miRNAs

LncRNAs primarily function as regulatory molecules through *cis*-acting mechanisms [30]. In the case of *cis*-acting regulation, lncRNAs regulate gene expression by interacting with neighboring protein-coding genes, which are identified as their potential target genes. Specifically, coding genes situated in a 10 kb region upstream and downstream of lncRNA genes were detected and designated as *cis*-regulatory targets of the corresponding lncRNAs [31]. In contrast, miRNAs exert their regulatory effects by binding to target sites via complementary base pairing. For miRNA target prediction, the 3′ untranslated region (3′ UTR) sequences of mRNAs from this species were used as candidate targets. MiRNA target gene prediction was performed using miRanda software [32].

### GO and KEGG functional enrichment analysis

To elucidate the infection mechanisms of *A. phagocytophilum*, the analyses of gene ontology (GO) annotation and Kyoto Encyclopedia of Genes and Genomes (KEGG) signaling pathway enrichment were conducted to investigate the functional roles dif-lncRNAs, dif-mRNAs, and dif-miRNAs [33, 34]. The GO database (http://www.geneontology.org) classifies functional annotations into three aspects: biological processes (BP), cellular components (CC), and molecular functions (MF). Similarly, KEGG (http://www.genome.jp/kegg/) enrichment analysis was further investigated for the potential significant pathways and assessed their potential biological significance. Both GO terms and KEGG pathways exhibited statistically significant enrichment at a threshold of *P* < 0.05.

### LncRNA–miRNA–mRNAA network construction

According to the competing endogenous RNA (ceRNA) hypothesis, lncRNAs and mRNAs regulate each other by competing for shared miRNA response elements (MREs) [35]. To elucidate the regulatory mechanisms of lncRNAs, miRNAs, and mRNAs in *A. phagocytophilum* infection, ceRNA interaction networks were established following the ceRNA hypothesis, incorporating differentially expressed transcripts (dif-lncRNAs, dif-miRNAs, and dif-mRNAs) [36]. The miRNA–mRNA and miRNA–lncRNA interactions were predicted using miRanda, and the resulting ceRNA network was visualized using the open-source software platform Cytoscape v3.5.1 [32, 37].

### Statistical analysis

Statistical analyses were performed using a paired Student’s *t*-test in GraphPad Prism version 8.0.2 (GraphPad Software, Inc., San Diego, CA, USA), and continuous data are expressed as mean ± standard deviation (SD). The relative expression levels of dif-lncRNAs, dif-mRNAs, and dif-miRNAs were calculated using the comparative 2^−△△Ct^ method, with the mean cycle threshold (Ct) values serving as the basis for calculation. All data were presented as mean ± standard deviation (SD). All experimental procedures were conducted with a minimum of three biological replicates. Results were considered statistically significant at* P* < 0.05.

## Results

### Influence of *A. phagocytophilum* infection on HL-60 cell apoptosis

**Fig. 1:**
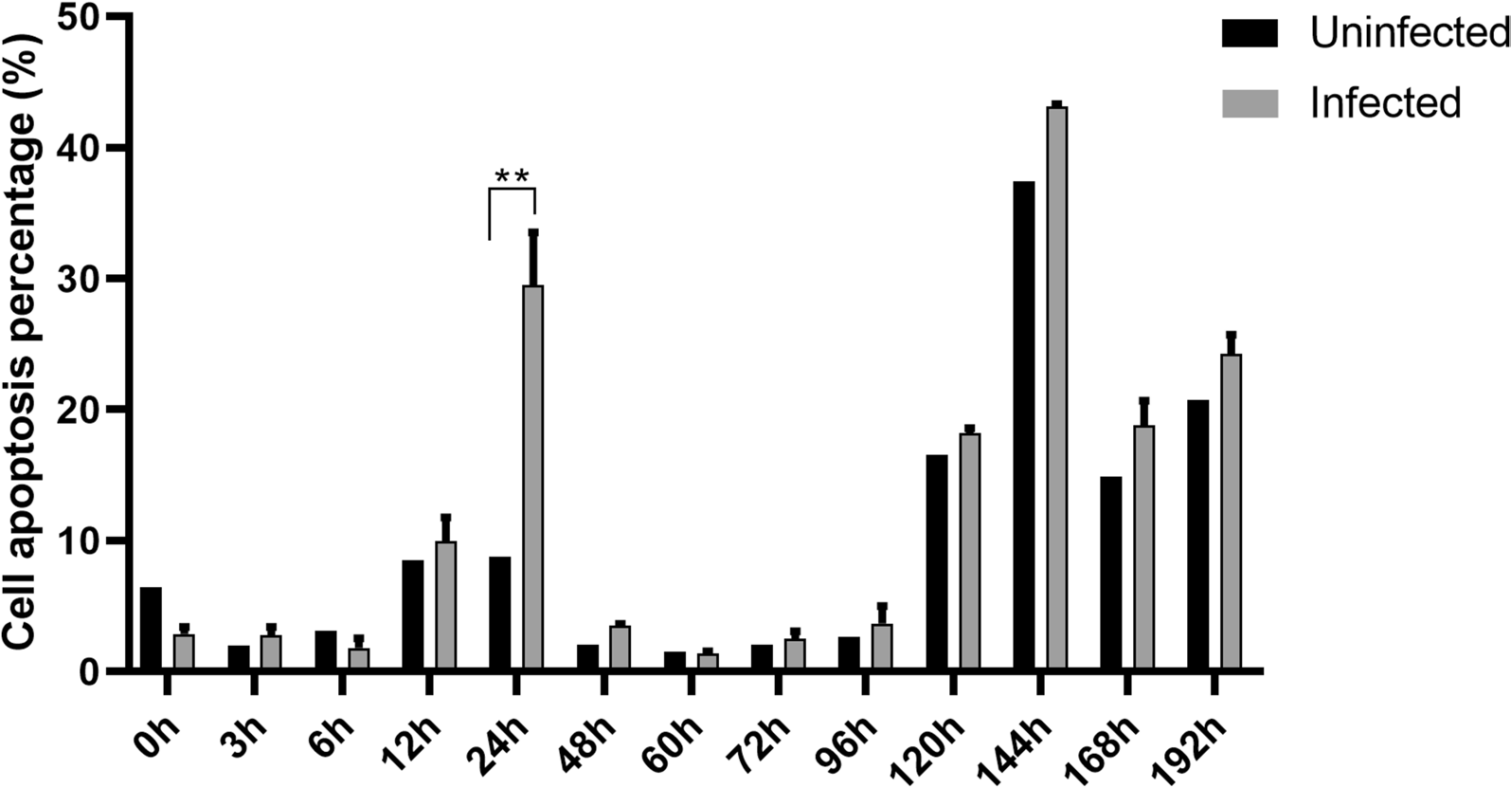
Apoptosis of HL-60 cells with or without *A. phagocytophilum* infection at various time points. Black bars represent uninfected control cells, and gray bars indicate infected cells. Asterisks denote statistically significant differences compared with the control group. ^*^P < 0.05; ^**^P = 0.0045

Apoptosis in HL-60 cells was evaluated by flow cytometry at multiple time points following *A. phagocytophilum* infection. The results showed that the apoptosis rate of *A. phagocytophilum*-infected HL-60 cells increased during the initial 3 h post-infection, followed by a deceleration at 6 h. A significant and sustained acceleration began at 12 h and persisted through 24 h, with apoptosis at 24 h significantly higher than in uninfected controls (*P* = 0.0045). Although apoptosis declined sharply between 24 and 48 h in both groups, it remained elevated in infected cells. Subsequently, uninfected cells exhibited a transient rise in apoptosis at 60 h, while infected cells maintained a consistently high apoptotic rate from 72 to 192 h. In summary, the apoptosis rate in infected HL-60 cells was generally higher than in uninfected controls from 0 to 192 h post-infection, except at the 6- and 60-h time points. However, among all time points examined, only the 24-h time point showed a statistically significant pro-apoptotic effect attributable to *A. phagocytophilum* infection (*P* < 0.05). The apoptosis rates of HL-60 cells with or without *A. phagocytophilum* infection at various time points are shown in Fig. 1, and the corresponding flow cytometric analysis is provided in Additional file 11: Fig. 7.

### Comprehensive whole transcriptome profiling

Raw sequencing data were filtered to remove low-quality reads, adapter contaminants, and sequences containing ambiguous bases (N) to ensure data quality. After processing, a total of 876,080,330 raw reads and 817,041,676 clean reads were generated from six cell samples during lncRNA library construction, averaging 12.90 Gb of clean reads per sample. The average Q30 and Q20 were 92.79% and 96.92%, respectively, indicating high-quality RNA-sequencing (RNA-seq) data. Similarly, during the construction of the miRNA library, a total of 108,765,043 raw reads and 82,583,913 clean reads were generated from the above cell samples, averaging 12.90 Gb of clean reads per sample, which further confirmed the high quality of the RNA-seq data.

### Profiling differentially expressed lncRNAs, mRNAs, and miRNAs

**Fig. 2:**
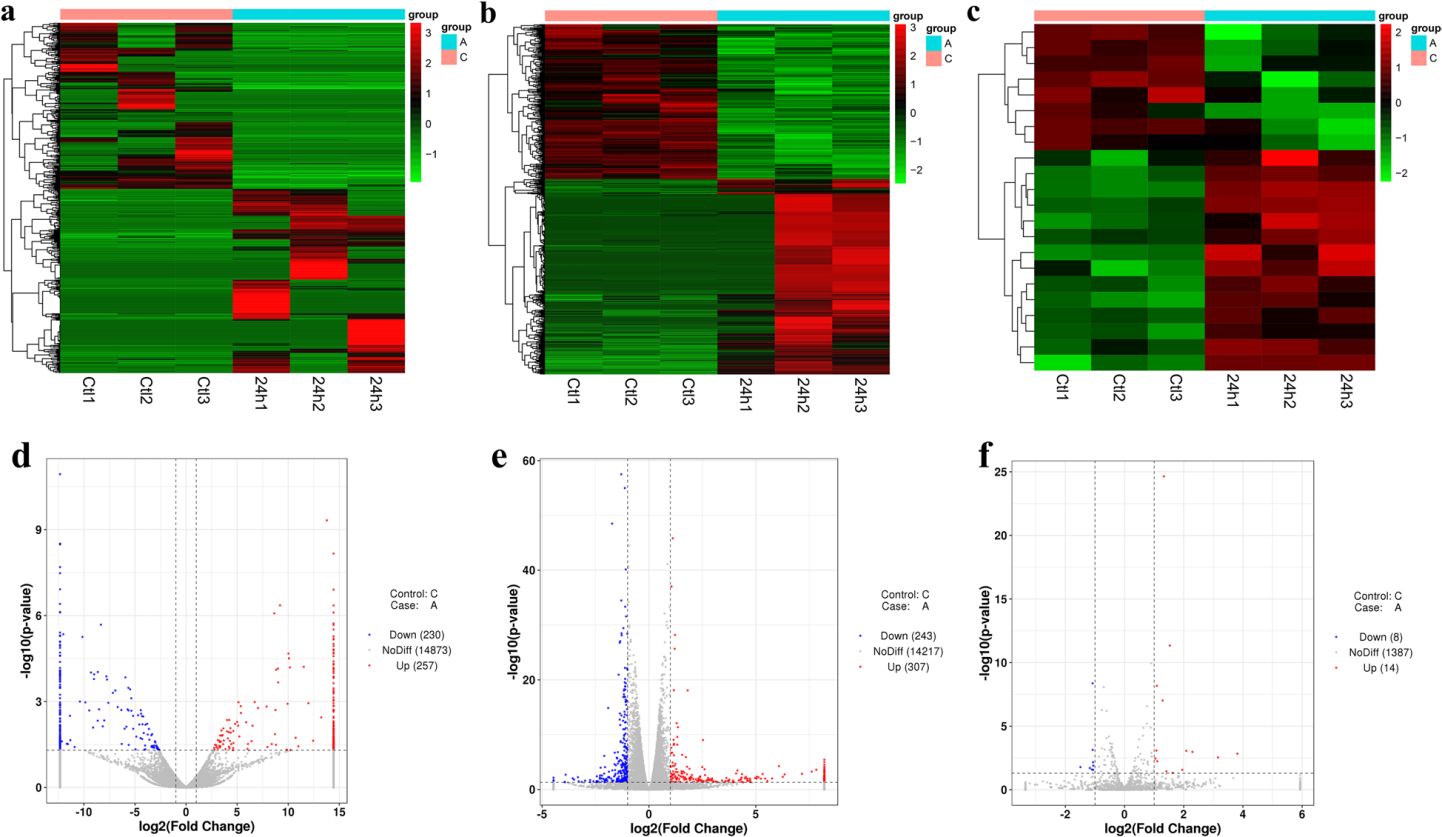
Both heatmaps and volcano plots analysis demonstrate differentially expressed molecules in HL-60 cells invaded by *A. phagocytophilum*. Partial heatmaps of dif-lncRNAs (**a**), dif-mRNAs (**b**), and dif-miRNAs (**c**) in HL-60 cells at 24 hpi. The volcano plots of dif-lncRNAs (**d**), the volcano plots of dif-mRNAs (**e**), and the volcano plots of dif-miRNAs (f) in HL-60 cells at 24 hpi. Red indicates upregulation, and green indicates downregulation

On the basis of the defined screening criteria, 487 lncRNAs (257 upregulated and 230 downregulated), 550 mRNAs (307 upregulated and 243 downregulated), and 22 miRNAs (14 upregulated and 8 downregulated) were identified as differentially expressed in HL-60 cells at 24 h hpi (Additional file 2: Table S2). The volcano plots and partial clustering analysis revealed distinct expression profiles for lncRNAs, mRNAs, and miRNAs, respectively (Fig. 2). As an additional assessment of sample similarity and replicate consistency, we included a sample-to-sample correlation heatmap in the Supplementary Information (Supplementary Fig. 1). The heatmaps demonstrate distinct clustering patterns that clearly segregate infected from control samples, thereby validating the differential expression analysis results.

### RT-qPCR validation assay

**Fig. 3:**
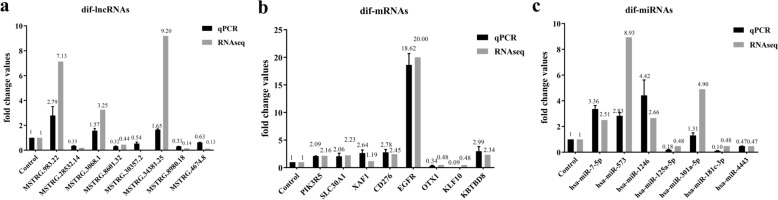
RT-qPCR validation of transcriptional changes 24 hpi. LncRNAs (**a**), mRNAs (**b**), and miRNAs (**c**) show consistent expression patterns with sequencing. Triplicate biological replicates (*n* = 3) were analyzed for each transcript, with gene names and normalized expression levels displayed on the horizontal and vertical axes, respectively

To confirm RNA-seq data reliability, eight lncRNAs (three upregulated, five downregulated), eight mRNAs (six upregulated, two downregulated), and seven miRNAs (four upregulated, three downregulated) were randomly selected for RT-qPCR validation. As demonstrated in Fig. 3, the expression levels of these validated genes exhibited strong consistency between the sequencing data and experimental validation results. Furthermore, lncRNA MSTRG.983.22, mRNA EGFR, and miRNA hsa-miR-1246 exhibited statistically significant differences relative to controls.

### Target gene prediction results for dif-lncRNAs and dif-miRNAs

By predicting the *cis*-regulated target genes of lncRNAs, the data demonstrated that a total of 59,953 *cis*-target genes were screened after *A. phagocytophilum* infection with HL-60 cells at 24 hpi (Additional file 3: Table S3).

By predicting the sequence matching values of miRNAs and mRNAs and predicting the potential target genes of differential miRNAs, the results showed that 22 miRNAs could target 34,313 mRNAs after *A. phagocytophilum* infection with HL-60 cells at 24 h (Additional file 4: Table S4).

### Functional enrichment analyses

**Fig. 4:**
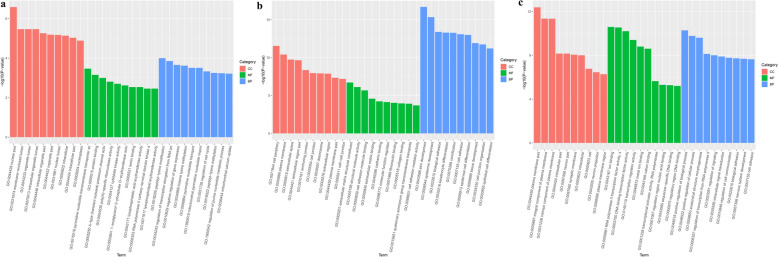
Functional enrichment analysis of RNA targets in HL-60 cells at 24 hpi. Bar graphs illustrating GO annotation analysis for targets associated with lncRNAs (**a**), mRNAs (**b**), and miRNAs (**c**) in HL-60 cells at 24 hpi

**Fig. 5:**
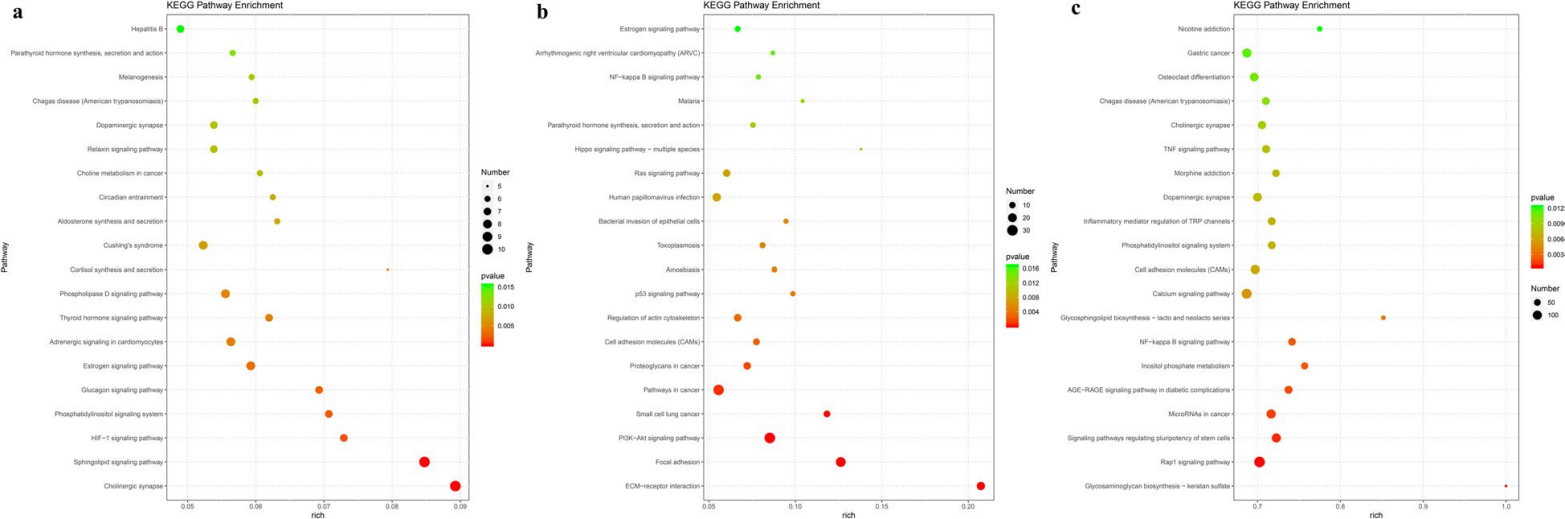
Analysis of the KEGG pathway. Top 20 significantly altered pathways mediated by dif-lncRNAs (**a**), dif-mRNAs (**b**) and dif-miRNAs (**c**) in HL-60 cells at 24 hpi. Pathway names are displayed on the *y*-axis, while gene ratios are shown on the *x*-axis. Circle size indicates the count of differentially expressed genes per pathway, while color intensity represents −log10 (*P*-value) ranges

The *cis*-regulated target genes of differentially expressed lncRNAs were subjected to functional enrichment analysis using the GO and KEGG databases. Functional enrichment analysis identified 883 significant GO terms at 24 hpi, including 684 in the biological process (BP) category (regulation of transcription elongation from RNA polymerase II promoter, negative regulation of gene expression, regulation of the cell cycle, and histone lysine methylation). Additionally, 81 were categorized under the cellular component (CC) category (nuclear part, membrane-enclosed lumen, intracellular organelle lumen, nuclear lumen, and intracellular part). Moreover, 118 were associated with the molecular function (MF) category [pyrimidine nucleotide transmembrane transporter activity, protein binding, A-type (transient outward) potassium channel activity, and poly(A)-specific ribonuclease activity] (see Additional file 5: Table S5 for details). The 30 most enriched GO terms (by significance) appear in Fig. 4. At 24 hpi, 56 biological pathways showed statistically significant enrichment, with notable examples including the phospholipase D signaling pathway, cortisol synthesis and secretion, and aldosterone biosynthesis (Additional file 6: Table S6). The top 20 most representative pathways in each group are shown in Fig. 5.

Functional categorization and enrichment profiling of differentially expressed mRNAs (dif-mRNAs) revealed statistically significant enrichment across 1398 gene ontology (GO) terms at 24 hpi. Biological process (BP) annotations dominated this dataset (1136 terms), primarily involving biological adhesion, cell adhesion, and cell junction organization. Cellular component (CC) terms (119 entries) localized predominantly to membrane-associated domains, including cell periphery, plasma membrane, and extracellular space. Molecular functions (MF) encompassed 143, comprising extracellular matrix structural constituent, structural molecule activity, cell adhesion molecule binding, and extracellular matrix binding (Additional file 7: Table S7). The 30 GO terms showing the highest enrichment significance appear in Fig. 4. Pathway analysis further identified 31 signaling pathways exhibiting statistically significant enrichment of dif-mRNAs at 24 hpi, notably featuring the PI3K–Akt signaling pathway, regulation of actin cytoskeleton, and p53 signaling pathway (comprehensive dataset available in Additional file 8: Table S8). The 20 most biologically significant pathways in each group are depicted in Fig. 5.

Functional characterization of predicted target genes associated with dif-miRNAs revealed statistically significant enrichment in 1417 GO terms at 24 hpi. The enriched GO terms demonstrated a distinct categorical distribution: Biological processes (BP) constituted the majority 1059 entries, primarily involving positive regulation of biological process, positive regulation of cellular process, regulation of transcription from RNA polymerase II promoter, and intracellular signal transduction. Cellular components (CC) included 157 membrane-centric annotations, such as the plasma membrane part, integral component of plasma membrane, intracellular part, intracellular, and cell. Molecular functions (MF) comprised 201 terms highlighting transcriptional regulation, including ion binding, RNA polymerase II transcription factor activity, sequence-specific DNA binding, DNA binding transcription factor activity, and metal ion binding (Additional file 9: Table S9). The 30 top-ranked GO terms by enrichment significance are visualized in Fig. 4. At 24 hpi, 48 signaling pathways exhibited significant enrichment, for example, the Rap1 signaling pathway and NF-κB signaling pathway (Additional file 10: Table S10). The 20 pathways showing the strongest enrichment significance in each category are illustrated in Fig. 5.

### Construction and comprehensive analysis of LncRNA–miRNA–mRNA network

**Fig. 6:**
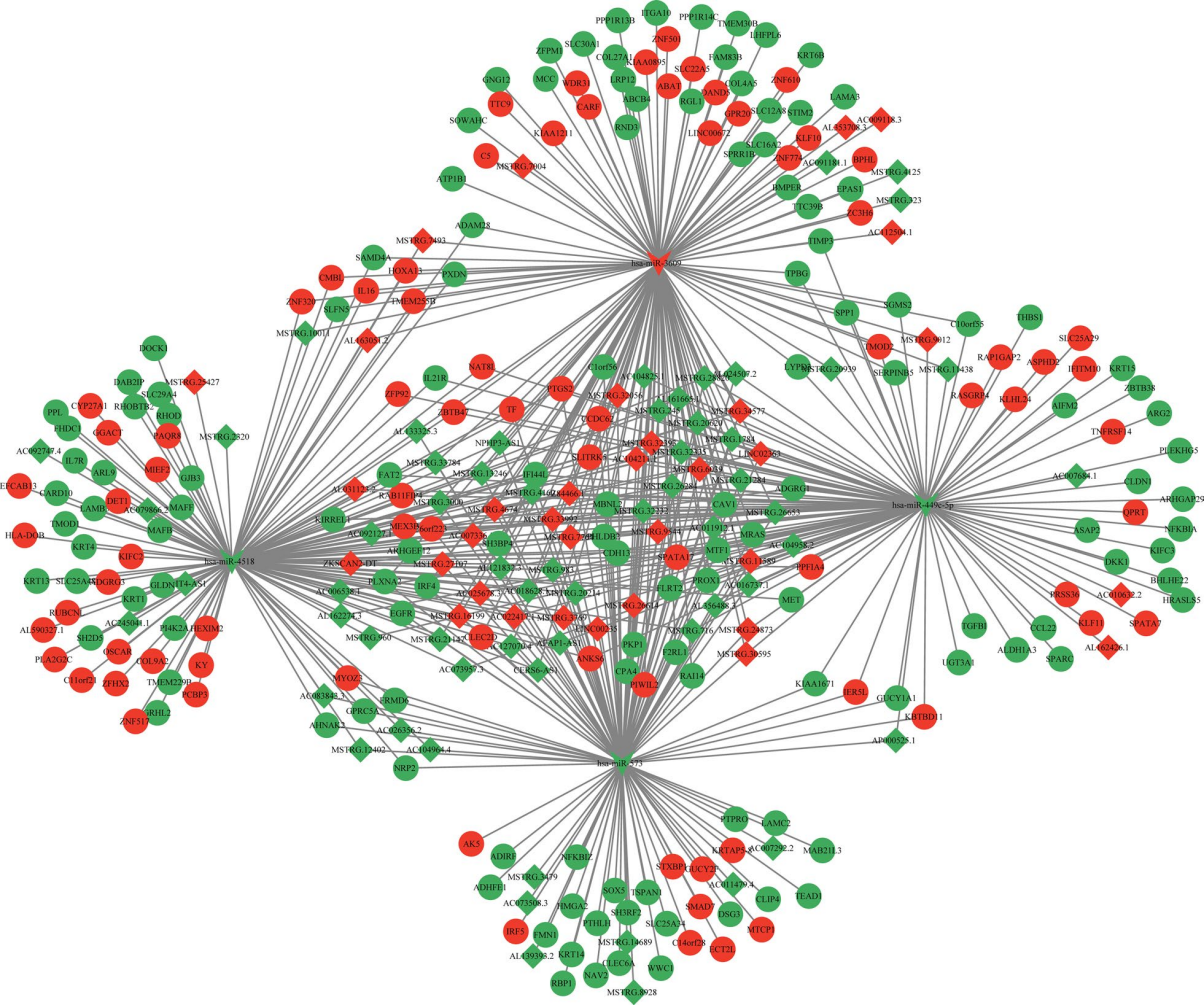
The competitive endogenous RNA interaction at 24 hpi. Green denote upregulated transcripts, and red shows downregulated genes. The diagram uses circular nodes (mRNA), *V*-shapes (miRNA), and diamonds (lncRNA) to represent different RNA types

LncRNAs absorb miRNAs, thereby inhibiting their binding to target mRNAs and demonstrating competitive endogenous RNA (ceRNA) functionality. The ceRNA hypothesis has expanded the biological roles of mRNAs and non-coding RNAs, attributing to them a broader range of functional significance. The construction of the ceRNA regulatory network has enabled a deeper understanding of the interactions between non-coding RNAs and mRNAs during *A. phagocytophilum* infection. The LncRNA–miRNA–mRNA network was constructed on the basis of the interactions between lncRNAs and miRNA targets, as well as mRNAs and miRNA targets. Target lncRNAs of miRNAs were predicted using miRanda software, while target mRNAs of miRNAs were identified using miRanda databases [32]. At 24 hpi in HL-60 cells invaded by *A. phagocytophilum*, a total of 311 LncRNA–miRNA–mRNA interactions were identified, comprising 94 lncRNAs, 213 mRNAs, and 4 miRNAs (hsa-miR-4518, hsa-miR-3609, hsa-miR-573, and hsa-miR-449c-5p) (Fig. 6). The findings reveal that multiple lncRNAs can simultaneously target miRNAs, and numerous mRNAs can be co-regulated, highlighting the critical genes contributing to *A. phagocytophilum* infection more efficiently.

## Discussion

The survival of *A. phagocytophilum* depends on host cell invasion, with neutrophils serving as its preferred site for replication and persistence [38]. However, the short lifespan of primary neutrophils poses significant challenges for in vitro studies, leading researchers to employ granulocytic cell lines such as HL-60, THP-1, and NB4 cells as alternative models [6, 39, 40]. Among these, differentiated HL-60 cells exhibit higher susceptibility to *A. phagocytophilum* infection compared with THP-1 and NB4 cells, accommodating up to 12 pathogens per cell, making them ideal for in vitro studies [41]. To investigate transcriptome changes in host cells following infection, we analyzed the expression patterns of mRNAs and non-coding RNAs (including miRNAs and lncRNAs) in HL-60 cells, comparing infected and control groups. The extensive differential expression of non-coding RNAs and mRNAs indicates complex regulatory mechanisms, including inhibition of apoptosis, modulation of inflammatory responses, and reprogramming of host metabolism that contribute to maintaining cellular homeostasis during *A. phagocytophilum* infection. Our findings diverged from those reported by Dumler et al. [42], which may result from host cell differentiation, all-*trans* retinoic acid (ATRA)-induced changes, or differences in sampling time points.

Previous studies of *A. phagocytophilum* global transcription profiles during key infection stages in ticks, human cell lines, and granulocytes have highlighted the crucial role of encoded proteins in establishing infection [22]. The pathogen suppresses apoptosis in human neutrophils by upregulating bfl-1, preserving mitochondrial membrane potential, and preventing caspase-3 activation [43]. In this research, we identified 550 differentially expressed mRNAs in infected HL-60 cells. The KEGG enrichment results highlighted three key pathways: PI3K-Akt signaling, actin cytoskeleton regulation, and p53 signaling. These pathways have been previously linked to *A. phagocytophilum* infection in megakaryocytic cells, though the specific roles of individual differentially expressed mRNAs require further investigation [44, 45].

The interaction between *A. phagocytophilum* and host microRNAs has emerged as a crucial regulatory mechanism. Previous studies have shown that the suppression of tick microRNA-133 leads to the upregulation of organic anion transporting polypeptide expression, which is essential for pathogen persistence and dissemination [20]. Additionally, *A. phagocytophilum* modulates the miRNA profiles of *I. scapularis* tick cells, particularly upregulating isc-mir-79 to promote infection through the Robo2 pathway [5]. Functional enrichment analyses of differentially expressed miRNA target genes using GO and KEGG databases identified significant enrichment in Rap1 and NF-κB signaling pathways, with hsa-miR-4518 and hsa-miR-3609 as central regulatory nodes within the complex ceRNA network, representing a novel direction for deciphering the molecular pathways in host–microbe interactions.

The differential expression of lncRNAs following *A. phagocytophilum* infection indicates significant molecular adaptations in HL-60 cells. Functional enrichment analysis revealed prominent involvement in the phospholipase D signaling pathway, cortisol synthesis and secretion, aldosterone synthesis, and secretion pathways. Elevated phosphatidylinositol diacylase (PLD) activity has been linked to the inhibition of apoptosis in cancer studies, suggesting a potential role for lncRNAs in mediating phospholipase-dependent responses during infection [46–49]. Increased cortisol and aldosterone secretion may contribute to host survival during physiological stress and intracellular calcium ion concentrations [50–57], thereby regulating various cellular processes. Collectively, these pathways may promote pathogen survival by suppressing apoptosis. While these steroid pathways are atypical in neutrophils/HL-60 cells, extra-adrenal glucocorticoid production occurs in activated immune cells [58, 59]. The findings probably indicate infection-driven crosstalk rather than local hormone synthesis, highlighting potential novel mechanisms worthy of future study.

Overall, these findings underscore the complex regulatory networks involved in host cell responses to *A. phagocytophilum* infection, particularly highlighting the biological significance of non-coding RNAs. Further exploration of these molecular interactions may reveal novel therapeutic targets for treating anaplasmosis and other related infectious diseases.

## Conclusions

This study represents the first comprehensive whole transcriptome profiling of *A. phagocytophilum*-infected HL-60 cells using RNA-seq technology. The results revealed significant differential expression of 550 mRNAs, 487 lncRNAs, and 22 miRNAs at 24 h post-infection (hpi), with 23 key RNAs independently validated by RT-qPCR. Functional enrichment analyses using GO and KEGG databases highlighted the prospective functions of these differentially expressed RNAs in the host’s cellular response to *A. phagocytophilum* invasion. Notably, the constructed ceRNA regulatory networks identified down-regulated hsa-miR-4518 and hsa-miR-3609 as central regulators of lncRNA and mRNA expression. These findings establish a foundation for future research into the complex molecular mechanisms of *A. phagocytophilum* infection, especially the pivotal functions of these core miRNAs and their interconnected regulatory circuits in mediating host–pathogen crosstalk. Understanding these mechanisms may ultimately facilitate the design of innovative treatment approaches for anaplasmosis.

## Supplementary Information

Supplementary Material 1. Table S1. Sequence-specific primer design for RT-qPCR analysis of miRNAs, mRNAs, and lncRNAs in HL-60 cells at 24 hpi

Supplementary Material 2. Table S2. Differential expression of mRNAs, lncRNAs, and miRNAs at 24 hpi

Supplementary Material 3. Table S3. Predicted *cis*-acting target genes for dif-lncRNAs at 24 hpi

Supplementary Material 4. Table S4. The target genes predicted for dif-miRNAs 24 hpi

Supplementary Material 5. Table S5. Functional Enrichment of *cis*-target genes for dif-lncRNAs at 24 hpi

Supplementary Material 6. Table S6. KEGG pathway enrichment in *cis*-targets of DE-lncRNAs at 24 hpi

Supplementary Material 7. Table S7. Functional enrichment of dif-mRNAs at 24 hpi

Supplementary Material 8. Table S8. KEGG pathway enrichment in dif-mRNAs at 24 hpi

Supplementary Material 9. Functional enrichment of DE-miRNA targets at 24 hpi

Supplementary Material 10. Table S10. KEGG pathway enrichment in target genes for dif-miRNAs at 24 hpi.

Supplementary Material 11. Fig. 7 Flow cytometric analysis of apoptosis in HL-60 cells with or without *A. phagocytophilum* infection over time. (a) Apoptosis rates of uninfected HL-60 cells at 0, 3, 6, 12, 24, 48, 60, 72, 96, and 144 h. (b) Apoptosis rates of *A. phagocytophilum*-infected HL-60 cells at the same time points under identical experimental conditions. Lower left: viable cells (Annexin V^−^/PI^−^); lower right: early apoptotic cells (Annexin V^+^/PI^−^); upper left: damaged cells (Annexin V^−^/PI^+^); upper right: late apoptotic and necrotic cells (Annexin V^+^/PI^+^)

Supplementary Material 12.

Supplementary Material 13. Fig. 8 Sample-to-sample correlation analysis shows the overall similarity and reproducibility of transcriptomic profiles in HL-60 cells invaded by *A. phagocytophilum* at 24 hpi. The correlation heatmap (a) displays pairwise correlation coefficients among control and 24 hpi samples based on the normalized expression matrix, with hierarchical clustering to visualize sample grouping (A: lncRNAs; B: mRNAs; C: miRNAs)

## Data Availability

All experimental results and derived data required to validate the conclusions are provided in the main text and supplementary materials. RNA-seq raw reads are available in National Center for Biotechnology Information (NCBI) Sequence Read Archive (SRA) (https://www.ncbi.nlm.nih.gov/sra) (BioProject range: SAMN43516315-SAMN43516320).

## Abbreviations

HL-60 cells: Human promyelocytic leukemia cells
RPMI: Roswell Park Memorial Institute
RBC: Red blood cell
BP: Biological process
CC: Cellular component
MF: Molecular function
ceRNA: Competing endogenous RNA
FC: Fold change
GO: Gene ontology
KEGG: Kyoto Encyclopedia of Genes and Genomes
RT-qPCR: Reverse transcription quantitative polymerase chain reaction

## References

[1] Read CB, Lind MCH, Chiarelli TJ, Izac JR, Adcox HE, Marconi RT, et al. The obligate intracellular bacterial pathogen *Anaplasma phagocytophilum* exploits host cell multivesicular body biogenesis for proliferation and dissemination. MBio. 2022;13:e0296122. 36409075 10.1128/mbio.02961-22 PMC9765717

[2] Mauri Pablo JD, Del Solar JJC, Hinojosa Enciso ET, Polveiro RC, Vieira DDS, Ramos Sanchez EM, et al. Anaplasmosis in the Amazon: diagnostic challenges, persistence, and control of *Anaplasma marginale* and *Anaplasma phagocytophilum*. Front Vet Sci. 2025;12:1571694. 40438413 10.3389/fvets.2025.1571694 PMC12116455

[3] Namjoshi P, Dahmani M, Sultana H, Neelakanta G. Rickettsial pathogen inhibits tick cell death through tryptophan metabolite mediated activation of p38 MAP kinase. iScience. 2022;26:105730. 36582833 10.1016/j.isci.2022.105730 PMC9792911

[4] Karshima SN, Ahmed MI, Kogi CA, Iliya PS. *Anaplasma phagocytophilum* infection rates in questing and host-attached ticks: a global systematic review and meta-analysis. Acta Trop. 2022;228:106299. 34998998 10.1016/j.actatropica.2021.106299

[5] Artigas-Jerónimo S, Alberdi P, Villar Rayo M, Cabezas-Cruz A, Prados PJE, Mateos-Hernández L, et al. *Anaplasma phagocytophilum* modifies tick cell microRNA expression and upregulates isc-mir-79 to facilitate infection by targeting the Roundabout protein 2 pathway. Sci Rep. 2019;9:9073. 31235752 10.1038/s41598-019-45658-2 PMC6591238

[6] Pedra JH, Sukumaran B, Carlyon JA, Berliner N, Fikrig E. Modulation of NB4 promyelocytic leukemic cell machinery by *Anaplasma phagocytophilum*. Genomics. 2005;86:365–77. 16005178 10.1016/j.ygeno.2005.05.008

[7] Rar V, Tkachev S, Tikunova N. Genetic diversity of *Anaplasma* bacteria: twenty years later. Infect Genet Evol. 2021;91:104833. 33794351 10.1016/j.meegid.2021.104833

[8] Wang F, Yan M, Liu A, Chen T, Luo L, Li L, et al. The seroprevalence of *Anaplasma phagocytophilum* in global human populations: a systematic review and meta-analysis. Transbound Emerg Dis. 2020;67:2050–64. 32180352 10.1111/tbed.13548

[9] Zhang L, Wang G, Liu Q, Chen C, Li J, Long B, et al. Molecular analysis of *Anaplasma phagocytophilum* isolated from patients with febrile diseases of unknown etiology in China. PLoS ONE. 2013;8:e57155. 23451170 10.1371/journal.pone.0057155 PMC3579781

[10] Yang J, Liu Z, Guan G, Liu Q, Li Y, Chen Z, et al. Prevalence of *Anaplasma phagocytophilum* in ruminants, rodents and ticks in Gansu, north-western China. J Med Microbiol. 2013;62:254–8. 23105025 10.1099/jmm.0.046771-0

[11] Zhu JJ, Zhang HZ, Hong RD, Yu D, Hong M, Liu ZX, et al. Prevalence and genetic diversity of *Anaplasma phagocytophilum* in wild small mammals from western Yunnan province, China. Front Vet Sci. 2024;11:1472595. 39539313 10.3389/fvets.2024.1472595 PMC11557534

[12] Ayllón N, Villar M, Galindo RC, Kocan KM, Šíma R, López JA, et al. Systems biology of tissue-specific response to *Anaplasma phagocytophilum* reveals differentiated apoptosis in the tick vector *Ixodes scapularis*. PLoS Genet. 2015;11:e1005120. 25815810 10.1371/journal.pgen.1005120 PMC4376793

[13] Ayllón N, Villar M, Busby AT, Kocan KM, Blouin EF, Bonzón-Kulichenko E, et al. *Anaplasma phagocytophilum* inhibits apoptosis and promotes cytoskeleton rearrangement for infection of tick cells. Infect Immun. 2013;81:2415–25. 23630955 10.1128/IAI.00194-13 PMC3697600

[14] Lee HC, Kioi M, Han J, Puri RK, Goodman JL. *Anaplasma phagocytophilum*-induced gene expression in both human neutrophils and HL-60 cells. Genomics. 2008;92:144–51. 18603403 10.1016/j.ygeno.2008.05.005

[15] de la Fuente J, Kocan KM, Blouin EF, Zivkovic Z, Naranjo V, Almazán C, et al. Functional genomics and evolution of tick-*Anaplasma* interactions and vaccine development. Vet Parasitol. 2010;167:175–86. 19819630 10.1016/j.vetpar.2009.09.019

[16] Chowdhury D, Choi YE, Brault ME. Charity begins at home: non-coding RNA functions in DNA repair. Nat Rev Mol Cell Biol. 2013;14:181–9. 23385724 10.1038/nrm3523 PMC3904369

[17] Liu J, Wang H, Chua NH. Long noncoding RNA transcriptome of plants. Plant Biotechnol J. 2015;13:319–28. 25615265 10.1111/pbi.12336

[18] Yang Y, Dai W, Sun Y, Zhao Z. Long non‑coding RNA linc00239 promotes malignant behaviors and chemoresistance against doxorubicin partially via activation of the PI3K/Akt/mTOR pathway in acute myeloid leukaemia cells. Oncol Rep. 2019;41:2311–20. 30720129 10.3892/or.2019.6991

[19] Bartel DP. MicroRNAs: genomics, biogenesis, mechanism, and function. Cell. 2004;116:281–97. 14744438 10.1016/s0092-8674(04)00045-5

[20] Ramasamy E, Taank V, Anderson JF, Sultana H, Neelakanta G. Repression of tick microRNA-133 induces organic anion transporting polypeptide expression critical for *Anaplasma phagocytophilum* survival in the vector and transmission to the vertebrate host. PLoS Genet. 2020;16:e1008856. 32614824 10.1371/journal.pgen.1008856 PMC7331985

[21] Yan Y, Lu C, Gong P, Pei Z, Peng Y, Jian F, et al. Molecular detection and phylogeny of *Anaplasma* spp. closely related to *Anaplasma phagocytophilum* in small ruminants from China. Ticks Tick Borne Dis. 2022;13:101992. 35777304 10.1016/j.ttbdis.2022.101992

[22] Nelson CM, Herron MJ, Wang XR, Baldridge GD, Oliver JD, Munderloh UG. Global transcription profiles of *Anaplasma phagocytophilum* at key stages of infection in tick and human cell lines and granulocytes. Front Vet Sci. 2020;7:111. 32211428 10.3389/fvets.2020.00111 PMC7069361

[23] Borjesson DL. Culture, isolation, and labeling of *Anaplasma phagocytophilum* for subsequent infection of human neutrophils. Methods Mol Biol. 2008;431:159–71. 18287755 10.1007/978-1-60327-032-8_13

[24] Cock PJ, Fields CJ, Goto N, Heuer ML, Rice PM. The Sanger FASTQ file format for sequences with quality scores, and the Solexa/Illumina FASTQ variants. Nucleic Acids Res. 2010;38:1767–71. 20015970 10.1093/nar/gkp1137 PMC2847217

[25] Kim D, Langmead B, Salzberg SL. HISAT: a fast spliced aligner with low memory requirements. Nat Methods. 2015;12:357–60. 25751142 10.1038/nmeth.3317 PMC4655817

[26] Langmead B. Aligning short sequencing reads with Bowtie. Curr Protoc Bioinform. 2010. 10.1002/0471250953.bi1107s32 PMC3010897 21154709

[27] Mortazavi A, Williams BA, McCue K, Schaeffer L, Wold B. Mapping and quantifying mammalian transcriptomes by RNA-Seq. Nat Methods. 2008;5:621–8. 18516045 10.1038/nmeth.1226 PMC13303166

[28] Zhou L, Chen J, Li Z, Li X, Hu X, Huang Y, et al. Integrated profiling of microRNAs and mRNAs: microRNAs located on Xq27.3 associate with clear cell renal cell carcinoma. PLoS ONE. 2010;5:e15224. 21253009 10.1371/journal.pone.0015224 PMC3013074

[29] Wang L, Feng Z, Wang X, Wang X, Zhang X. DEGseq: an R package for identifying differentially expressed genes from RNA-seq data. Bioinformatics. 2010;26:136–8. 19855105 10.1093/bioinformatics/btp612

[30] Yan P, Luo S, Lu JY, Shen X. *Cis*- and trans-acting lncRNAs in pluripotency and reprogramming. Curr Opin Genet Dev. 2017;46:170–8. 28843809 10.1016/j.gde.2017.07.009

[31] Gil N, Ulitsky I. Regulation of gene expression by *cis*-acting long non-coding RNAs. Nat Rev Genet. 2020;21:102–17. 31729473 10.1038/s41576-019-0184-5

[32] John B, Enright AJ, Aravin A, Tuschl T, Sander C, Marks DS. Human microRNA targets. PLoS Biol. 2004;2:e363. 15502875 10.1371/journal.pbio.0020363 PMC521178

[33] Thomas PD. The gene ontology and the meaning of biological function. Methods Mol Biol. 2017;1446:15–24. 27812932 10.1007/978-1-4939-3743-1_2 PMC6438694

[34] Kanehisa M, Furumichi M, Tanabe M, Sato Y, Morishima K. KEGG: new perspectives on genomes, pathways, diseases and drugs. Nucleic Acids Res. 2017;45:D353–61. 27899662 10.1093/nar/gkw1092 PMC5210567

[35] Salmena L, Poliseno L, Tay Y, Kats L, Pandolfi PP. A ceRNA hypothesis: the Rosetta Stone of a hidden RNA language? Cell. 2011;146:353–8. 21802130 10.1016/j.cell.2011.07.014 PMC3235919

[36] Tay Y, Rinn J, Pandolfi PP. The multilayered complexity of ceRNA crosstalk and competition. Nature. 2014;505:344–52. 24429633 10.1038/nature12986 PMC4113481

[37] Otasek D, Morris JH, Bouças J, Pico AR, Demchak B. Cytoscape automation: empowering workflow-based network analysis. Genome Biol. 2019;20:185. 31477170 10.1186/s13059-019-1758-4 PMC6717989

[38] Schaff UY, Trott KA, Chase S, Tam K, Johns JL, Carlyon JA, et al. Neutrophils exposed to *A. phagocytophilum* under shear stress fail to fully activate, polarize, and transmigrate across inflamed endothelium. Am J Physiol Cell Physiol. 2010;299:C87-96. 20392928 10.1152/ajpcell.00165.2009 PMC2904253

[39] Garcia-Garcia JC, Barat NC, Trembley SJ, Dumler JS. Epigenetic silencing of host cell defense genes enhances intracellular survival of the rickettsial pathogen *Anaplasma phagocytophilum*. PLoS Pathog. 2009;5:e1000488. 19543390 10.1371/journal.ppat.1000488 PMC2694362

[40] Carlyon JA, Chan WT, Galán J, Roos D, Fikrig E. Repression of rac2 mRNA expression by *Anaplasma phagocytophila* is essential to the inhibition of superoxide production and bacterial proliferation. J Immunol. 2002;169:7009–18. 12471136 10.4049/jimmunol.169.12.7009

[41] Rennoll-Bankert KE, Sinclair SH, Lichay MA, Dumler JS. Comparison and characterization of granulocyte cell models for *Anaplasma phagocytophilum* infection. Pathog Dis. 2014;71:55–64. 24376092 10.1111/2049-632X.12111 PMC4037391

[42] Dumler JS, Sinclair SH, Shetty AC. Alternative splicing of differentiated myeloid cell transcripts after infection by *Anaplasma phagocytophilum* impacts a selective group of cellular programs. Front Cell Infect Microbiol. 2018;8:14. 29456968 10.3389/fcimb.2018.00014 PMC5801399

[43] Ge Y, Yoshiie K, Kuribayashi F, Lin M, Rikihisa Y. *Anaplasma phagocytophilum* inhibits human neutrophil apoptosis via upregulation of bfl-1, maintenance of mitochondrial membrane potential and prevention of caspase 3 activation. Cell Microbiol. 2005;7:29–38. 15617521 10.1111/j.1462-5822.2004.00427.x

[44] Khanal S, Sultana H, Catravas JD, Carlyon JA, Neelakanta G. *Anaplasma phagocytophilum* infection modulates expression of megakaryocyte cell cycle genes through phosphatidylinositol-3-kinase signaling. PLoS ONE. 2017;12:e0182898. 28797056 10.1371/journal.pone.0182898 PMC5552339

[45] Cabezas-Cruz A, Alberdi P, Valdes JJ, Villar M, de la Fuente J. Remodeling of tick cytoskeleton in response to infection with *Anaplasma phagocytophilum*. Front Biosci. 2017;22:1830–44. 10.2741/4574 28410148

[46] Kopp F, Mendell JT. Functional classification and experimental dissection of long noncoding RNAs. Cell. 2018;172:393–407. 29373828 10.1016/j.cell.2018.01.011 PMC5978744

[47] Su W, Chen Q, Frohman MA. Targeting phospholipase D with small-molecule inhibitors as a potential therapeutic approach for cancer metastasis. Future Oncol. 2009;5:1477–86. 19903073 10.2217/fon.09.110 PMC2814819

[48] Kang DW, Choi KY, Min S. Phospholipase D meets Wnt signaling: a new target for cancer therapy. Cancer Res. 2011;71:293–7. 21224347 10.1158/0008-5472.CAN-10-2463

[49] Esteller M. Non-coding RNAs in human disease. Nat Rev Genet. 2011;12:861–74. 22094949 10.1038/nrg3074

[50] Pierouli K, Papageorgiou L, Mitsis T, Papakonstantinou E, Diakou I, Leptidis S, et al. Role of microRNAs and long non‑coding RNAs in glucocorticoid signaling (review). Int J Mol Med. 2022;50:147. 36367164 10.3892/ijmm.2022.5203 PMC9662139

[51] Reiisi S, Ahmadi K. Bioinformatics analysis of a disease-specific lncRNA-miRNA-mRNA regulatory network in recurrent spontaneous abortion (RSA). Arch Gynecol Obstet. 2024;309:1609–20. 38310583 10.1007/s00404-023-07356-3

[52] Bao M, Li H, Li J. Identification of potential lncRNA-miRNA-mRNA regulatory network contributing to aldosterone-producing adenoma. J Cell Mol Med. 2022;26:5614–23. 36305047 10.1111/jcmm.17586 PMC9667512

[53] Butterworth MB. Non-coding RNAs and the mineralocorticoid receptor in the kidney. Mol Cell Endocrinol. 2021;521:111115. 33301840 10.1016/j.mce.2020.111115 PMC7796954

[54] Leimena C, Qiu H. Non-coding RNA in the pathogenesis, progression and treatment of hypertension. Int J Mol Sci. 2018;19:927. 29561765 10.3390/ijms19040927 PMC5979335

[55] Hanxiao Y, Boyun Y, Minyue J, Xiaoxiao S. Identification of a novel competing endogenous RNA network and candidate drugs associated with ferroptosis in aldosterone-producing adenomas. Aging. 2023;15:9193–216. 37709486 10.18632/aging.205028 PMC10522391

[56] Huang W, Cane MC, Mukherjee R, Szatmary P, Zhang X, Elliott V, et al. Caffeine protects against experimental acute pancreatitis by inhibition of inositol 1,4,5-trisphosphate receptor-mediated Ca^2+^ release. Gut. 2017;66:301–13. 26642860 10.1136/gutjnl-2015-309363 PMC5284483

[57] Skogestad J, Aronsen JM, Tovsrud N, Wanichawan P, Hougen K, Stokke MK, et al. Coupling of the Na^+^/K^+^-ATPase to Ankyrin B controls Na^+^/Ca^2+^ exchanger activity in cardiomyocytes. Cardiovasc Res. 2020;116:78–90. 30949686 10.1093/cvr/cvz087

[58] Keresztes M, Horváth T, Ocsovszki I, Földesi I, Serfőző G, Boda K, et al. ACTH- and cortisol-associated neutrophil modulation in coronary artery disease patients undergoing stent implantation. PLoS ONE. 2013;8:e71902. 23967262 10.1371/journal.pone.0071902 PMC3743772

[59] Taves MD, Gomez-Sanchez CE, Soma KK. Extra-adrenal glucocorticoids and mineralocorticoids: evidence for local synthesis, regulation, and function. Am J Physiol Endocrinol Metab. 2011;301:E11-24. 21540450 10.1152/ajpendo.00100.2011 PMC3275156

