# Supplementary material for: Whole transcriptome analysis reveals differential gene expression associated with Anaplasma phagocytophilum invading HL-60 cells
Source: Parasit Vectors. 2026 May 8;19:290. doi: 10.1186/s13071-026-07381-6 (PMC13366880; doi:10.1186/s13071-026-07381-6)
Supplement: Supplementary file 1 — Supplementary Material 1. Table S1. Sequence-specific primer design for RT-qPCR analysis of miRNAs, mRNAs, and lncRNAs in HL-60 cells at 24 hpi [file 13071_2026_7381_MOESM1_ESM.docx]

**Additional file 1: Table S1.** Primers designed for qRT-PCR validation of candidate lncRNAs, mRNAs and miRNAs in HL-60 cells at 24 hour post infection with *A. phagocytophilum*.

| **Genes** | **Primers** |  |
| --- | --- | --- |
|  | **Forward** | **Reverse** |
| MSTRG.983 | GAGGAAGGAGCCACAGGTAGGG | AGAGGCAAGGACTGGAGGGAAG |
| MSTRG.28532 | CCACCCTCCCGACTTCCTAGC | GCACCCAGACAACTACAGCACAG |
| MSTRG.3068 | AGGAGGGACTAGGTGAAGGACAG | ATGGGTAGAGGCAGCTCAGGTG |
| MSTRG.8601 | GAAACGGTGGCTGGAGGGATAATG | GCAGGGAAGGGACAGGGAAGG |
| MSTRG.30357 | GGCACAGCAAGCAAGGTCTAGG | AAGAGGAGGAAGCAGGAAGGAAGG |
| MSTRG.34381 | TGTCTCTGTGCCCCAACCAAAAC | CACCCTCACTTCCAATCCCAACTG |
| MSTRG.8980 | CTCCAGCCCTTGTCCTCCCTTC | ACACAGTCACGGAATGCCAGAATC |
| MSTRG.4674 | GGCAGGAGAATGGCGTGAACC | GGAATCTCACTCTGTCACCCAAGC |
| PIK3R5 | CCACACTTCCCACCAGACTC | ACTTCCACTGGGTTCAGCAG |
| SLC30A1 | TTCATGTTCATGGTGCTGGAGGTG | AGCATGTGGAAGGAGTCGGAGAG |
| XIA | GCTTACTGCCTGCGGTTCCTG | ATGCTCTGCTGACACATCGTACAC |
| CD276 | ATGCTCTGCTGACACATCGTACAC | CTCCTGTGAGGCAGAACCAC |
| EGFR | GCCTTGACTGAGGACAGCAT | AATCTGCCACTGTTTCCCCC |
| OTX1 | ACCACCCGTTGAGCCAGTCC | AAGCCCAGAGCCACCGTAGC |
| KLF10 | GCAGCCAGCATCCTCAACTATCAG | TGACACAGCGGCACATGGTATG |
| KBTBD8 | TCCTTCCATCCAAGACCAATGTGC | TCGATCTCCGAGTTCCTGATGACC |
| hsa-miR-7-5p | GCGTGGAAGACTAGTGATTTTGTTGT | GCTGTCAACGATACGCTACGTAACG |
| hsa-miR-573 | CGCTGAAGTGATGTGTAACTGATCAG | GCTGTCAACGATACGCTACGTAACG |
| hsa-miR-1246 | GCGCAATGGATTTTTGGAGCAGG | GCTGTCAACGATACGCTACGTAACG |
| hsa-miR-125a-5p | CTCCCTGAGACCCTTTAACCTGTGA | GCTGTCAACGATACGCTACGTAACG |
| hsa-miR-301a-5p | GCTCTGACTTTATTGCACTACT | GCTGTCAACGATACGCTACGTAACG |
| hsa-miR-181c-3p | AACCATCGACCGTTGAGTGGAC | GCTGTCAACGATACGCTACGTAACG |
| hsa-miR-4443 | CGTTGGAGGCGTGGGTTTT | GCTGTCAACGATACGCTACGTAACG |
| *GAPDH* | CCACATCGCTCAGACACCAT | CCAGGCGCCCAATACG |
| *β-actin* | AGCGAGCATCCCCCAAAGTT | GGGCACGAAGGCTCATCATT |
| *U6* | GCTTCGGCAGCACATATACAAAAT | CGCTTCACGAATTTGCGTGTCAT |
