# Supplementary figures and images for: Whole transcriptome analysis reveals differential gene expression associated with Anaplasma phagocytophilum invading HL-60 cells
Source: Parasit Vectors. 2026 May 8;19:290. doi: 10.1186/s13071-026-07381-6 (PMC13366880; doi:10.1186/s13071-026-07381-6)

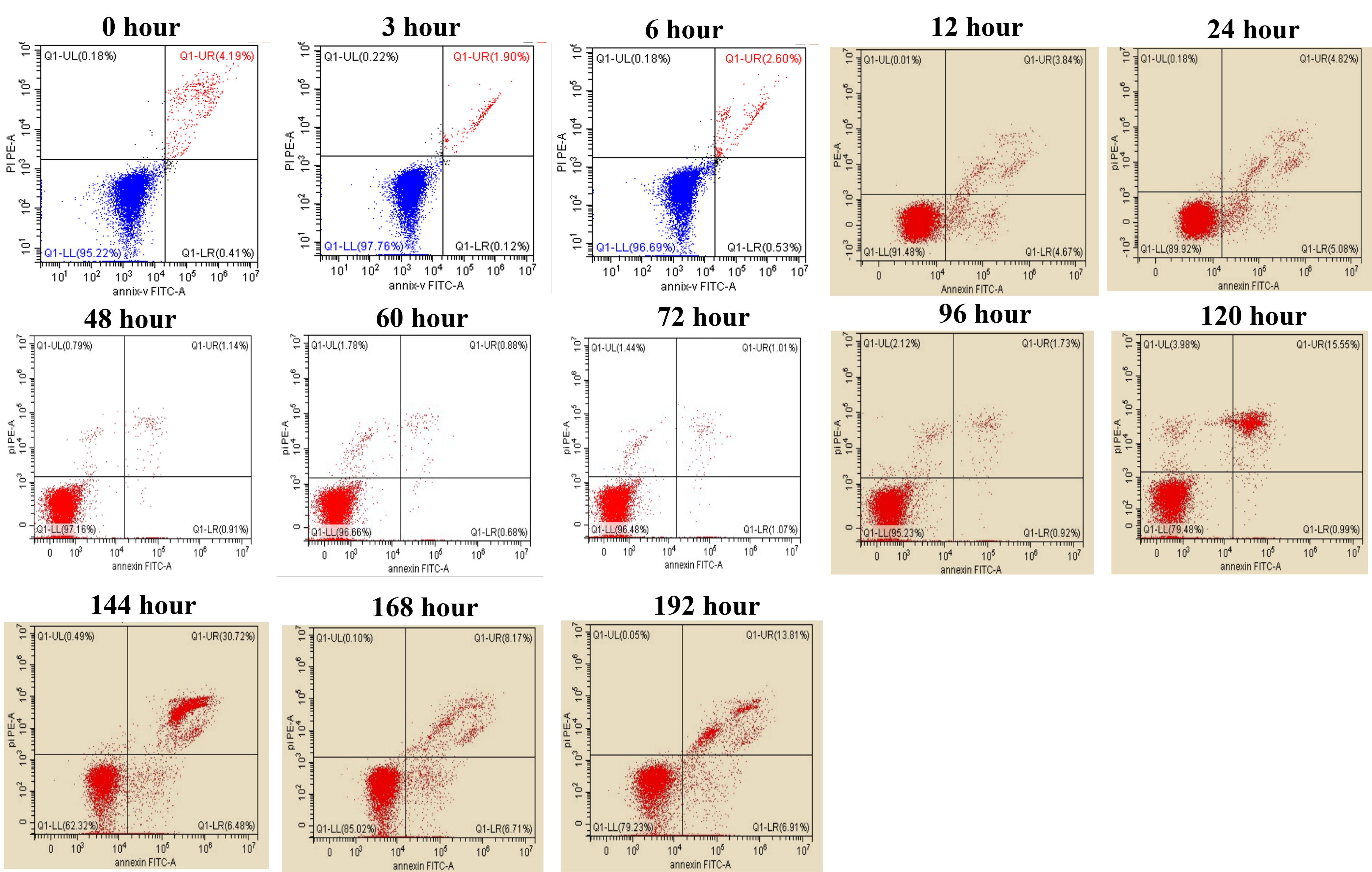

Supplement: Supplementary file 11 — Supplementary Material 11. Fig. 7 Flow cytometric analysis of apoptosis in HL-60 cells with or without A. phagocytophilum infection over time. (a) Apoptosis rates of uninfected HL-60 cells at 0, 3, 6, 12, 24, 48, 60, 72, 96, and 144 h. (b) Apoptosis rates of A. phagocytophilum-infected HL-60 cells at the same time points under identical experimental conditions. Lower left: viable cells (Annexin V−/PI−); lower right: early apoptotic cells (Annexin V+/PI−); upper left: damaged cells (Annexin V−/PI+); upper right: late apoptotic and necrotic cells (Annexin V+/PI+) [file 13071_2026_7381_MOESM11_ESM.tif]

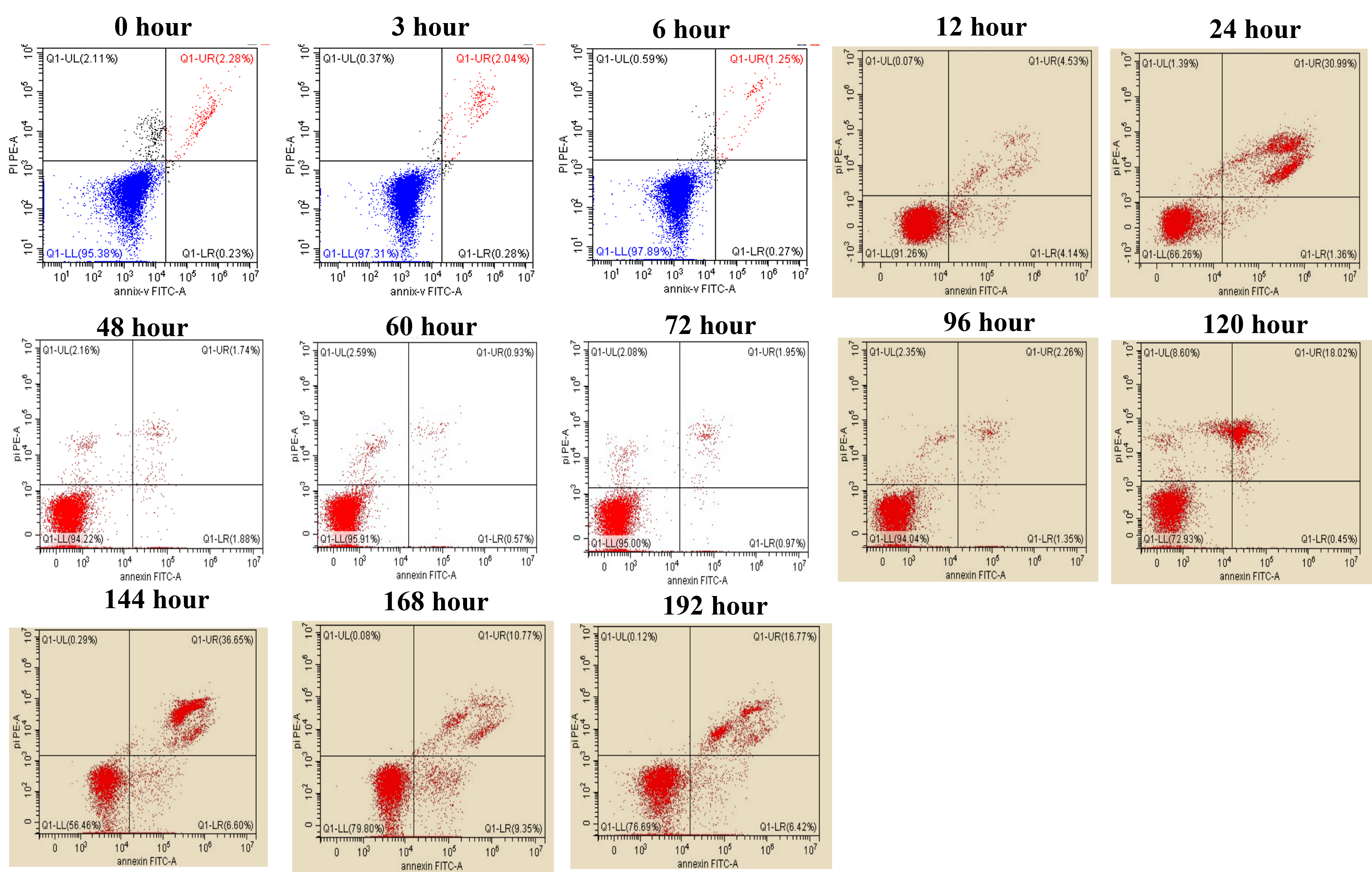

Supplement: Supplementary file 12 — Supplementary Material 12. [file 13071_2026_7381_MOESM12_ESM.tif]

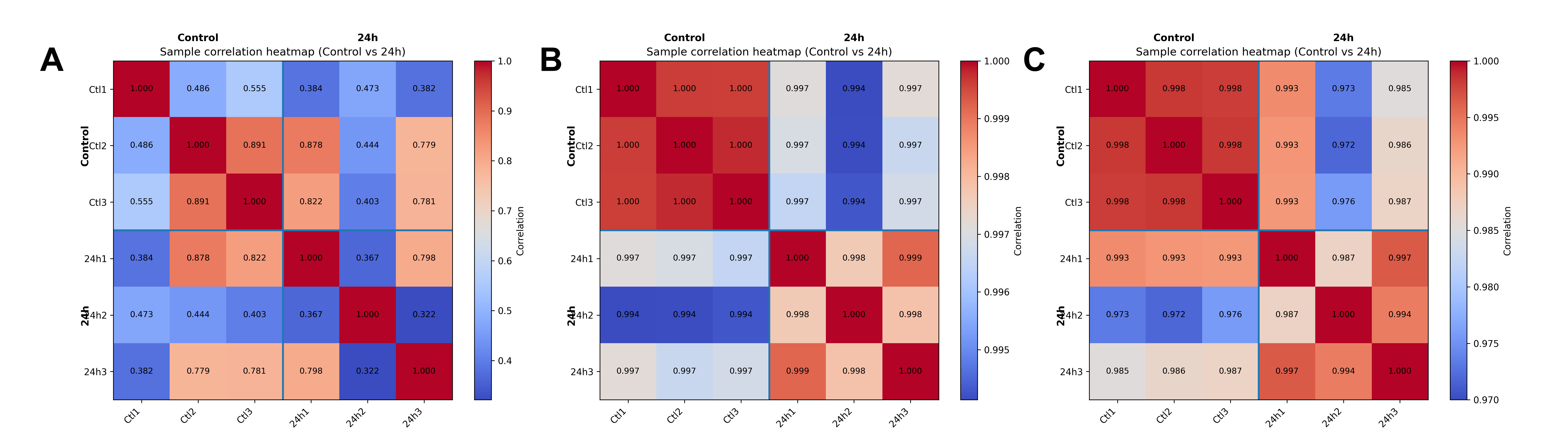

Supplement: Supplementary file 13 — Supplementary Material 13. Fig. 8 Sample-to-sample correlation analysis shows the overall similarity and reproducibility of transcriptomic profiles in HL-60 cells invaded by A. phagocytophilum at 24 hpi. The correlation heatmap (a) displays pairwise correlation coefficients among control and 24 hpi samples based on the normalized expression matrix, with hierarchical clustering to visualize sample grouping (A: lncRNAs; B: mRNAs; C: miRNAs) [file 13071_2026_7381_MOESM13_ESM.tif]
